# Lassa Fever in Pregnancy: Report of 2 Cases Seen at the University College Hospital, Ibadan

**DOI:** 10.1155/2016/9673683

**Published:** 2016-03-09

**Authors:** O. O. Bello, O. R. Akinajo, K. H. Odubamowo, T. A. O. Oluwasola

**Affiliations:** ^1^Department of Obstetrics & Gynaecology, University College Hospital, PMB 5116, Ibadan, Nigeria; ^2^Department of Obstetrics & Gynaecology, College of Medicine, University of Ibadan, Ibadan, Nigeria

## Abstract

Lassa fever (LF), an acute viral haemorrhagic fever, is an endemic zoonotic viral infection in West Africa countries with up to 15% case fatality rate. Though a rodent-borne infection, it can also be transmitted from person to person during the care of sick relations or more commonly in health care settings as a nosocomial infection. Vertical transmission from mother to child has been documented. We report 2 cases of LF among pregnant women which were managed at the University College Hospital, Ibadan, between September and October 2014. Both patients were in their early 20s with only one surviving the disease. Both had supportive therapy but none had antiviral therapy. This report emphasized the importance of early presentation, high index of suspicion, prompt diagnosis, and early commencement of supportive therapy in the management of patients suspected with LF especially in the era of other viral haemorrhagic infections.

## 1. Introduction

Lassa fever (LF) is an acute viral haemorrhagic illness caused by Lassa virus, a member of the virus family Arenaviridae. LF was first recognized in 1969 in Lassa, Northern Nigeria, and has since become endemic especially in West Africa countries like Sierra Leone, Guinea, Mali, Liberia, and Nigeria [1–6]. It is of great public health concern such that a single case is referred to as an outbreak. There are estimated 300,000–500,000 cases of Lassa fever each year [3–5] with a mortality rate of 15%–20% for hospitalized patients, which can become as high as 50% during epidemics and up to 90% in third-trimester pregnancies for both the expectant mother and fetus [4, 6]. Presently, there is no licensed vaccine or immunotherapy available for prevention or treatment of this disease and although the antiviral drug ribavirin is somewhat beneficial, it must be administered at an early stage of infection to successfully alter disease outcome, thereby limiting its utility. In addition, there is no commercially available Lassa fever diagnostic assay, which hampers early detection and rapid implementation of existing treatment regimens (e.g., ribavirin administration) [3–6].

The documented prevalence of antibodies to the virus in Africa has varied between 7% in Guinea [2], 8–52% in Sierra Leone [6], and 21% in Nigeria [7]. The main mode of spread is from infected rodents to man although it can equally be transmitted from person to person commonly through ingestion and inhalation but also through exchange of body fluids in a Lassa virus infected individual, during the care of sick relations or in health care settings [1]. Secondary human spread can result from percutaneous or permucosal exposure to blood and other infected body fluids, especially if the fluids contain blood. Studies have shown that person-to-person transmission of this virus contributes less than rodent contact to human infection [8] and this type of transmission can be minimized by timely infection-control measures, careful management of infected patients, and, in some cases, administration of personal protective equipment (PPE) to health care workers [1, 9].

Transmission peaks in the acute phase of fever when the virus is present in the throat while in the early stage its symptoms and signs are indistinguishable from those of other common conditions in the tropics such as malaria, typhoid, and other viral haemorrhagic fevers [10]. The symptoms occur 1–3 weeks after the exposure though it could be asymptomatic thereby making the clinical diagnosis difficult [1, 3–6]. About 80% of Lassa fever virus infections symptoms are mild and are undiagnosed but death may occur within two weeks after symptom onset due to multiorgan failure [1].

LF can be diagnosed by immunofluorescent antibody (IFA) or enzyme linked immunosorbent assay (ELISA) by detecting an increase in antibody titer or the presence of specific IgM and IgG as well as Lassa antigen [1, 9]. Reverse transcription-polymerase chain reaction (RT-PCR) can be used in the early stage of disease [1]. In facilities with high containment laboratories, the virus can be cultured in 7–10 days while immunohistochemistry performed on formalin-fixed specimens can be used to make a postmortem diagnosis [1, 9, 11]. There are currently commercially available rapid diagnostic kits that are effective in facilitating early diagnosis and prompt management [4].

In the absence of vaccine, LF can be prevented by keeping rats away from homes and rapidly investigating a suspected case which will help in facilitating prompt response in terms of treatment, implementation of isolation measures, using PPE, contact tracing, and the education of people in high-risk areas on ways of reducing rodent populations in their homes [11–13]. Supportive treatment in form of fluid and electrolyte management is often necessary.

In pregnancy, Lassa virus causes high maternal and fetal mortality due to high viral concentrations in the maternal blood, placenta, and fetal tissue [4, 7, 14]. Good maternal outcomes have been previously documented [4] but spontaneous abortion complicates the infection with an estimated 95% mortality in fetuses of infected pregnant mothers, 92% in early pregnancy, 75% in the third trimester, and 100% in the neonatal period for term babies [1, 7, 14]. Maternal mortality is 7% in the first two trimesters, 30% in the last trimester, and 50% within one month of delivery compared to less than 15% in the general population of nonpregnant women being 13% [7, 11, 14].

The outbreak of LF in a community within Ibadan at a time between 2014 and 2015 when some West African countries were plagued with Ebola viral disease (another viral haemorrhagic disease with similar symptoms) made it pertinent to highlighting the challenges of care and management for these two pregnant women in our facility.

## 2. Case Presentation

*Case  1*. Mrs. AA, 23-year-old gravida 2, para 1 (1 alive) trader, presented on September 16, 2014, at the gestational age of 18 weeks and 5 days with 5-day history of fever and 12-hour history of altered level of consciousness. There was associated history of jaundice, vomiting, and mild abdominal pain. There was no history of ingestion of herbal concoction. She had no cough, skin reaction, haematuria, or diarrhea. History of travel or contact was negative. She had no known intercurrent illness. At examination, she was icteric and dehydrated but neither pale nor febrile. She also had nontender hepatomegaly (6 cm below the right costal margin) but other parameters were essentially normal.

An initial assessment was that of fulminant hepatitis with encephalopathy to consider acute fatty liver of pregnancy and exclude viral haemorrhagic fever. Laboratory investigations were essentially normal except for markedly deranged liver enzymes (alkaline phosphatase, ALP, 176 iu, aspartate aminotransferase, AST, 2877 iu, and alanine aminotransferase, ALT, 2229 iu, with elevated direct and indirect bilirubin). Hepatitis and human immunodeficiency virus (HIV) screening were negative while the obstetric ultrasound was essentially normal for the gestational age.

In the absence of rapid diagnostic kits, samples were obtained for serology and RT-PCR for Lassa and Ebola viruses while she was comanaged with the gastroenterologist. She had repeated seizures with associated dyspnea and worsening coma scale. Viral haemorrhagic screening test was positive for Lassa virus and negative for Ebola but unfortunately further management was hampered by nonavailability of space in the intensive care unit.

She remained on supportive therapy till the third day when she had cardiopulmonary arrest with failed attempt at resuscitation. Consent for autopsy was declined by the relatives on religious grounds but necessary control and surveillance measures were ensured on the first-line contacts.

*Case  2*. Mrs. OB, 24-year-old nulliparous legal practitioner, presented at the accident and emergency department of our facility on October 2, 2014, at the gestational age of 12 weeks with 2-day history of continuous high grade fever, body weakness, vomiting, and diarrhea though the review of the systems was essentially normal. About 10 days before the onset of symptoms, she had visited a part of Ibadan for one week which was later confirmed to have an outbreak of LF. She was conscious and alert with no associated constitutional symptoms.

Laboratory studies including the liver function parameters were within the normal limits in this particular patient. Viral screening was however positive for Lassa virus but negative for Ebola virus. She was nursed on the isolation ward and all her vital signs remained within normal limits except the temperature which was intermittently high but resolved after 2 days. The patient and her husband declined ribavirin based on the fear of its risks. They requested transferring their care to Lagos and have since been lost to follow-up.

## 3. Discussion

In 2012, there was an outbreak of Lassa fever in Nigeria in which 623 cases and 70 deaths were recorded [15]. In Ibadan, between September and November 2014, there were 80 suspected cases of viral haemorrhagic fever managed in our hospital, out of which 27 were positive for Lassa fever.

The first case presented in this study presented in an advanced stage of the disease which was evidenced by the development of complications such as loss of consciousness and seizure disorders. While waiting for the result of the viral screening, she was commenced on supportive therapy. Unfortunately, she died before the result was available and therefore could not be commenced on antiviral therapy. The antiviral drug of choice, ribavirin, is effective in the treatment of Lassa fever but only if administered early in the course of illness [1, 3–6]. In a study of Lassa fever in Sierra Leone, it was observed that patients with a high risk of death who were treated for 10 days with intravenous ribavirin, begun within the first six days after the onset of fever, had a case fatality rate of 5% while patients whose treatment began seven or more days after the onset of fever had a case fatality rate of 26% [16]. In another study it was reported that Lassa fever carries a high risk to the fetus throughout pregnancy and to the mother, especially in the third trimester with a documented maternal death of 20% due to Lassa fever [14, 15].

The second case presented in the hospital at the early stage of the infection and with supportive therapy she improved clinically. Surviving LF without antiviral treatment has been documented but rare [17].

A case report written previously suggested that patients with laboratory confirmed Lassa fever who have episodes of falling temperature with low level of AST (usually <150 iu per liter) on admission and improving clinical state may not require treatment with ribavirin [17]. This further emphasized the importance of early presentation, high index of suspicion by the health workers, commencement of supportive therapy, and prompt diagnosis of Lassa fever. Elevated levels of interleukin 6 have been documented as biomarker for poor prognosis but its assay is yet to be commenced in our centre. However, ensuring an effective vaccine against LF will remain a desirable step [18].

## 4. Conclusion

These reports emphasized the importance of early presentation, high index of suspicion among health workers, and prompt diagnosis of patients with Lassa fever so as to reduce the mortality rate of this infection in our area. They also suggest that conservative management of symptoms of the disease and its complications may improve survival of individuals with this infection. Availability of rapid diagnostic kit will significantly contribute positively to good outcome for the patients if it can be made available in our hospitals.
